# Kinetics of SARS-CoV-2 Spike Antibodies after the Second and Third Dose of the BNT162b2 COVID-19 Vaccine and Association with Epidemiological Characteristics and Breakthrough Infection in a Cohort Study of Healthcare Workers

**DOI:** 10.3390/microorganisms11082010

**Published:** 2023-08-04

**Authors:** Elizabeth-Barbara Tatsi, Filippos Filippatos, Charilaos Dellis, Maria-Myrto Dourdouna, Vasiliki Syriopoulou, Athanasios Michos

**Affiliations:** 1 Infectious Diseases and Chemotherapy Research Laboratory, First Department of Pediatrics, Medical School, ‘Aghia Sophia’ Children’s Hospital, National and Kapodistrian University of Athens, 11527 Athens, Greece; etatsi@med.uoa.gr (E.-B.T.); vsyriop@med.uoa.gr (V.S.); 2 University Research Institute of Maternal and Child Health and Precision Medicine, 11527 Athens, Greece

**Keywords:** SARS-CoV-2, COVID-19, BNT162b2, immunity, vaccination, breakthrough infection

## Abstract

To prospectively study the kinetics of immune responses after immunization with the BNT162b2 mRNA COVID-19 vaccine and their association with epidemiological parameters and breakthrough infection (BI), we measured total (TAbs-WT) and neutralizing antibodies against wild-type (NAbs-WT) and Omicron (NAbs-O) SARS-CoV-2 spike proteins in healthcare workers (HCWs) after the second (4 and 8 months) and third dose (1 and 8 months). Vaccinated HCWs (*n* = 486), with a median age (IQR) of 49 years (38–56), were included in this prospective cohort study. BI was observed 4 and 8 months after the second dose in 8/486 (1.6%) and 15/486 (3.1%) HCWs, respectively, and 1 and 8 months after the third dose in 17/486 (3.5%) and 152/486 (31.3%) HCWs, respectively. A comparison of immune responses 1 month after the third dose in vaccinated HCWs without a BI or with a BI in the next 7 months did not detect any statistically significant differences in the TAbs-WT (median (IQR): 16,611.0 (13,011.0) U/mL vs. 17,572.5 (14,501.0) U/mL, *p* = 0.529) and NAbs-WT (median (IQR): 96.5% (1.7) vs. 96.7% (1.9), *p* = 0.555). After infection, HCWs with a BI had significantly increased TAbs-WT levels at all time points compared to healthy HCWs. The findings of the present study indicate that antibody levels after three doses of the BNT162b2 vaccine are not directly associated with the possibility of a BI.

## 1. Introduction

The COVID-19 pandemic accelerated the production of vaccines to prevent and control the pandemic [1]. The most widely used mRNA vaccine for COVID-19 is BNT162b2 (Pfizer/BioNTech), which was first released in January 2021 and approved by the US Food and Drug Administration (FDA) in August 2021 [2]. This vaccine includes the full-length mRNA of the SARS-CoV-2 spike protein that induces the humoral (including neutralizing antibodies) and cellular host immune response. The most important region in the spike protein is the receptor-binding domain (RBD), which binds with the human receptor, angiotensin-converting enzyme 2 (ACE2), in order to enter and infect the host cell [3,4]. The fundamental blocking role of RBD-ACE2 binding is due to the neutralizing antibodies. An interesting hypothesis is that CD147 plays a role as an alternative receptor of the spike SARS-CoV-2 protein and as a potential therapeutic target, as it has been described in other infections such as malaria [5].

Several studies have examined the duration of antibody levels after two vaccine doses, revealing their rapid decline [6,7]. Before the end of 2021, a third booster dose with the BNT162b2 COVID-19 vaccine was recommended by the national vaccination committee. In the same period, a new variant of SARS-CoV-2 called Omicron emerged, which shortly replaced the Delta variant and is still circulating now. The multiple mutations in the spike protein in the Omicron variant allow this variant to evade the immune response that has been developed due to previous infection or vaccination [8]. A recent study that investigated the immune response six months after the administration of a booster dose of an mRNA vaccine revealed that antibody levels are declining, but are still at a high level [9]. In the post-COVID-19 era, despite the high rate of the immunized population, reinfections and breakthrough infections (BIs) are continuously being recorded, increasing the anxiety of a possible new, contagious variant [10,11].

However, there are many unanswered questions, as long-term evidence regarding the kinetics of antibody levels after the second and third dose of the BNT162b2 mRNA COVID-19 vaccine with epidemiological parameters and breakthrough infections are limited.

The aim of this study was to investigate the association of total and neutralizing antibodies against the receptor-binding domain (RBD) of the SARS-CoV-2 spike protein with epidemiological and clinical parameters in a cohort of healthcare workers (HCWs) after the second and third dose of the BNT162b2 vaccine, as well as to estimate the number of breakthrough infection cases and correlate them with epidemiological data.

## 2. Materials and Methods

### 2.1. Study Design and Participants

In this prospective cohort study, healthcare workers (HCWs) were included from the “Aghia Sophia” Children’s Hospital, which is the largest tertiary pediatric hospital in Athens, Greece. The HCWs were healthcare professionals (medical doctors, nurses, and technicians) and nonmedical personnel of the hospital who were vaccinated against the SARS-CoV-2 virus with two and three doses of the Pfizer/BioNTech BNT162b2 vaccine in January–February 2021 and April–August 2022, respectively.

The participants were voluntarily tested for their humoral adaptive immune response to the SARS-CoV-2 vaccine. Blood sampling was performed at four time points for all participants: four and eight months after the second dose and one and eight months after the third dose (Figure S1). The determination of the seropositivity and neutralization activity of antibodies was conducted in the Infectious Diseases Laboratory, First Department of Pediatrics, Medical School, “Aghia Sophia” Children’s Hospital, National and Kapodistrian University of Athens.

A form for collecting demographic and clinical data as well as adverse events (AEs) after the third vaccine dose was completed by each participant. A detailed description of the epidemiological form was presented in a previous publication [7].

For the design of the study, we used the STROBE checklist (Table S1) [12], where the primary outcome was total SARS-CoV-2 spike protein antibodies and the secondary outcome was the neutralization activity of SARS-CoV-2 antibodies against the wild type and Omicron variant.

The study protocol was approved by the scientific and bioethics committee of “Aghia Sophia” Children’s Hospital (No. 2794/10 February 2021) and informed consent was obtained from all participants.

### 2.2. Total Antibody Detection against SARS-CoV-2

Serum samples were tested using the Elecsys Anti-SARS-CoV-2 S (Roche Diagnostics, Basel, Switzerland) reagent on a Cobas e 411 immunoassay analyzer for the semiquantitative detection of total antibodies (TAbs-WT: IgA, IgM and IgG) against the S1 subunit of the wild-type SARS-CoV-2 spike protein, according to the manufacturer’s instructions. This assay is an Electrochemiluminescence Immunoassay (ECLIA), which is based on a double-antigen sandwich Enzyme-Linked Immunosorbent Assay (ELISA) methodology. The sensitivity of this assay, as reported in the product brochure, is 98.8% (95% CI: 98.1–99.3%) and values ≥ 0.8 U/mL are positive.

### 2.3. Neutralization Assays against SARS-CoV-2 Wild Type and Omicron Variant

The determination of neutralizing antibodies (NAbs) against the SARS-CoV-2 wild type (NAbs-WT) and Omicron variant (NAbs-O) was conducted using the cPass^TM^ SARS-CoV-2 Neutralization Antibody Detection kit (GenScript Biotech Corporation, Piscataway, NJ, USA) only in vaccinated HCWs without BI, according to the manufacturer’s instructions. This kit is based on a blocking ELISA using a horseradish peroxidase (HRP)-conjugated recombinant SARS-CoV-2 RBD fragment for the WT and Omicron variant and the human angiotensin-converting enzyme 2 (ACE2) receptor protein. The optical density (OD) was measured at 450 nm with the Labtech LT4500 microtiter plate reader, and the percentage of RBD-specific neutralization antibodies was calculated by the following equation: Percentage signal inhibition (%) = (1 − OD value of sample/OD value of negative control) × 100. Percentages ≥ 30% were considered positive.

### 2.4. Statistical Methods

Absolute and relative frequencies (%) were used to describe the qualitative variables such as demographic characteristics, while mean, standard deviation (SD), median, and interquartile range (IQR) were used for quantitative data. Differences between two or more independent samples were assessed with the Mann–Whitney U test or Kruskal–Wallis H test, respectively. Post hoc analysis was performed using Tukey–Kramer’s test. Also, two-way analysis of variance (ANOVA) was used. The assumption of normality was checked through kurtosis and skewness, Kolmogorov–Smirnov, and Shapiro–Wilk tests. The statistical significance level was set as a *p*-value ≤ 0.05. Statistical analysis was performed using SPSS version 26.0 (IBM Corp., released 2019. IBM SPSS Statistics for Windows, Version 26.0. Armonk, NY, USA: IBM Corp).

## 3. Results

### 3.1. Study Population

A cohort of 486 vaccinated HCWs with three doses of the BNT162b2 COVID-19 vaccine and a median age of 49 (IQR: 38–56) years was included in the study. In total, 77.6% (377/486) were female. Out of the entire study population, the most common adverse events after the third dose were local pain (63.6%, 294/462) and fatigue (29.2%, 135/462) (Table S2). Non-severe adverse events were reported.

Breakthrough infections were recorded on the individual history forms and confirmed through antibody measurements, or in the case of asymptomatic HCWs, the BI was directly verified via the increase in antibody levels. The HCWs with a BI were separately analyzed. Demographic and epidemiological data from both groups, which consist of vaccinated HCWs with and without a BI, after each vaccine dose are shown in Table 1.

### 3.2. Breakthrough Infections

During the study period, breakthrough SARS-CoV-2 infections were detected in 192/486 (39.5%) immunized HCWs. Specifically, four and eight months after the second dose, a BI happened in 8/486 (1.6%) and 15/486 (3.1%) HCWs, respectively. The median time interval between the BI and administration of the second vaccine dose was 6 (IQR: 3–9) months. Asymptomatic BI cases occurred in 4/23 (17.4%) HCWs, while 9/23 (39.1%) reported symptoms without testing for SARS-CoV-2.

Among the HCWs with a confirmed BI (*n* = 10), the median duration of symptoms was 3 (IQR: 0–6) days. The median number of reported symptoms was 3 (IQR: 1–7), with the most common being nasal congestion (*n* = 7), anosmia (*n* = 6), ageusia (*n* = 4), fatigue (*n* = 4), and myalgias/arthralgias (*n* = 4).

One and eight months after the third dose, a BI happened in 17/486 (3.5%) and 152/486 (31.3%) HCWs, respectively, with a median interval between the BI and the administration of the third dose of 4 (IQR: 3–5) months. There were 32 (18.9%) asymptomatic cases, while 41 (24.3%) HCWs reported symptoms without SARS-CoV-2 testing.

Among the HCWs with a confirmed BI (*n* = 96), the median duration of symptoms was 4 (IQR: 0.5–9.5) days. The median number of reported symptoms (*n* = 138) was 3 (IQR: 1.8–5), with the most common being nasal congestion (*n* = 90), fatigue (*n* = 81), cough (*n* = 71), pharyngalgia (*n* = 67), headache (*n* = 54), and myalgias/arthralgias (*n* = 36).

### 3.3. Antibody Kinetics after Second and Third BNT162b2 COVID-19 Vaccine

Antibody levels after the second and third doses of the BNT162b2 COVID-19 vaccine are presented in Table 2. In vaccinated HCWs without a BI, a significant decrease in TAbs-WT levels was detected eight months compared to four months after the second vaccine dose (median (IQR): 413.1 (479.6) U/mL vs. 703.6 (716.1) U/mL, *p*-value <0.001) as well as eight months compared to one month after the third dose (median (IQR): 4441.5 (6037.0) U/mL vs. 17,241.5 (14,479.0) U/mL, *p*-value <0.001). In contrast, TAbs-WT levels of HCWs with a BI were increased at both eight-month points (median (IQR): 6551.0 (14,909.0) U/mL in second dose and 25,000.0 (9498.0) U/mL in the third dose) compared to four months (median (IQR): 5017.5 (11,185.4) U/mL) and one month (median (IQR): 24,863.5 (8671.5) U/mL) after each vaccine dose (*p*-value <0.001). At all time points, HCWs with a BI had significantly elevated TAbs-WT compared to HCWs without a BI (*p*-value < 0.05, Table 2).

**Figure 1:**
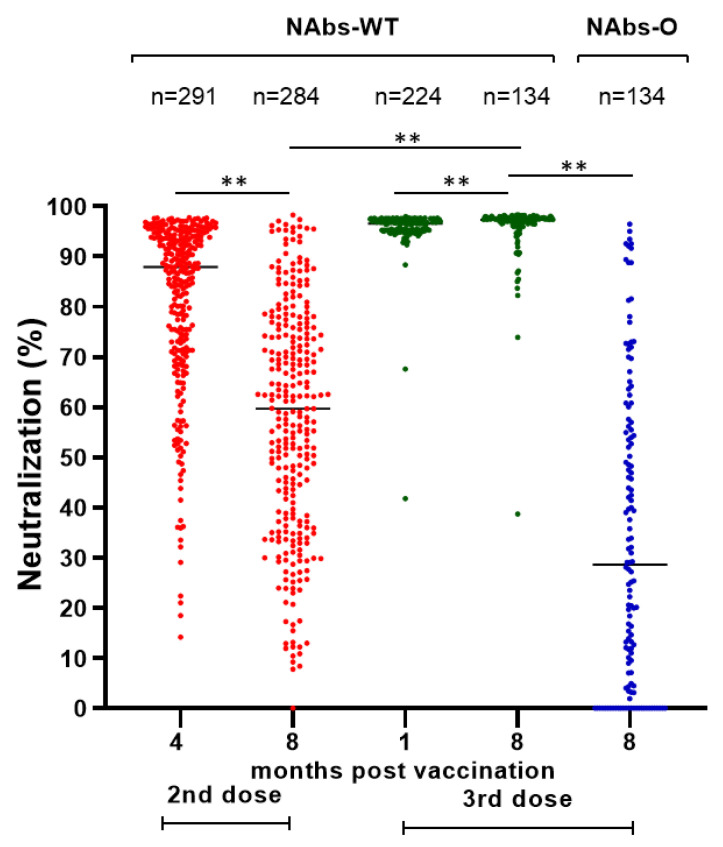
Neutralizing antibodies (%) against SARS-CoV-2 spike protein of wild type (NAbs-WT) and Omicron variant (NAbs-O) after second (4 and 8 months) and third (1 and 8 months) doses of BNT162b2 vaccine in HCWs without breakthrough infection. Notes: NAbs-WT after the second and third dose is marked in red and green, respectively. NAbs-O is marked in blue. ** *p*-value < 0.001 for Mann–Whitney U test.

A similar decline was detected in the NAbs-WT levels of vaccinated HCWs without a BI after the second vaccine dose (median (IQR): 87.70% (22.20) at four months and 59.70% (37.30) at eight months), while after the third dose there was a slight but significant increase in NAbs-WT levels (median (IQR): 96.60% (1.80) at one month and 97.30% (1.40) at eight months) (Figure 1). After the second dose, 1.7% (5/291) and 9.9% (28/284) of HCWs had NAbs-WT levels < 30% after four and eight months, respectively. After the third dose, NAbs-WT levels < 30% were not detected. Eight months after the third dose, only 47.8% (64/134) of HCWs had NAbs-O >30%.

A comparison of immune responses one month after the third dose between vaccinated HCWs without a BI or with a BI in the next 7 months did not detect any statistically significant differences in the TAbs-WT (median (IQR): 17,572.5 (14,501.0) U/mL vs. 16,611.0 (13,011.0) U/mL, *p*-value =0.529) and NAbs-WT levels (median (IQR): vs. 96.7% (1.9), 96.5% (1.7), *p*-value = 0.555).

### 3.4. Association of Antibody Levels with Epidemiological Data

Statistical analysis of the epidemiological data of HCWs without a BI, which covered their immune response at each time point, detected many different interactions (Table 3). A significant interaction was found between sex and NAbs-WT one month after the third dose (*p*-value = 0.016), with males having a higher NAbs-WT than females (median (IQR): 96.9% (2.3) vs. 96.5% (1.8), respectively).

Statistical significance was also found in TAbs-WT and NAbs-WT after the second dose and in TAbs-WT one month after the third dose, depending on the age group of the HCWs and smoking status, as presented in Table 3. Antibody levels were inversely related to age. For example, eight months after the second dose the age group ≥ 60 years old (TAbs-WT median (IQR): 269.0 (326.2) U/mL and NAbs-WT median (IQR): 45.2% (35.0)) had fewer antibodies compared to the 20–29-year-old group (TAbs-WT median (IQR): 716.5 (507.3) U/mL and NAbs-WT median (IQR): 66.5% (27.0)).

Smokers are characterized by lower antibody levels; for example, eight months after the second dose the median (IQR) values of TAbs-WT and NAbs-WT were 327.3 (420.7) U/mL and 50.8% (35.4) in smokers compared to 437.0 (494.6) U/mL and 62.3% (34.3) in non-smokers.

Autoimmune diseases (ADs) were found to negatively interact with the TAbs-WT (median (IQR): 3518.0 (4475.0) U/mL in HCWs with AD vs. 4550.5 (7490.0) U/mL in HCWs without AD) and NAbs-O levels [median (IQR): 15.9% (31.8) in HCWs with AD vs. 34.9% (52.9) in HCWs without ADs] eight months after the third dose (*p*-values = 0.043 and 0.049, respectively). However, when we compared all of the points together, the interaction remained significant only for the TAbs-WT levels (*p*-values = 0.029). There was not a statistically significant difference in immune responses regarding underlying diseases at any time point during the study.

The corresponding analysis in HCWs with a BI found only one significant interaction between Tabs-WT levels and sex (*p*-value = 0.027, Table S3). Age groups, smoking status, and autoimmune and underlying diseases did not affect the immune responses of this cohort.

## 4. Discussion

In the present study, we investigated the duration of the humoral immune response after the second and third dose of the BNT162b2 COVID-19 vaccine, the rate of BI, and their correlation with the epidemiological data. There was a high rate of BI detected eight months after the third dose. These findings indicate that the levels of antibodies after three doses of the BNT162b2 vaccine are not directly associated with the possibility of a BI.

In the present study, injection-site pain and fatigue were the most common adverse events after the administration of a third BNT162b2 vaccine dose. In concordance with our results are other large cohort studies from Europe and Asia in which local pain and fatigue were also found to be the most common adverse events after the third dose [13,14,15]. A recent English study investigated the severity of adverse events after the administration of the BNT162b2 third dose in healthy HCWs and HCWs with a previous COVID-19 infection and had a similar cohort size with our study, revealing that the common adverse events were the same in both groups. Further, healthy HCWs reported more events than HCWs with a previous COVID-19 infection (before the third vaccine dose), which is similar to the findings of this study [13].

It is known that the antibody response after vaccination wanes [16]; however, the rate of decline, the level of protection against infection and severe illness, and the parameters that are affected are still under investigation. Compared to our previous study in the same cohort, the antibody response (TAbs-WT and NAbs-WT) after the third dose was much more elevated compared to that after previous vaccine doses [7]. Similar results have been reported by other studies [17,18].

Interestingly, although a small proportion of HCWs were negative for NAbs-WT after the second dose at all time points, none were negative after the third dose, revealing the necessity of this booster dose to protect against severe illness. Studies like ours also detected a low production of neutralizing antibodies against the Omicron variant, even in individuals with special diseases like cancer patients [19]. However, it remains unknown whether they play a protective role [18,20].

Before the administration of a third dose and the emergence of the Omicron SARS-CoV-2 variant, the rate of BI was very low (<10%) up to 7 months after the booster [16,20,21]. In this study, BIs were also detected after the second dose in a low percentage and one month after the third dose, while a rapid increase in BI cases (36.5%) was identified eight months after the third dose. A similar rate of BI (41.9%) was reported in a recent study 6 months after the booster dose [18]. On the contrary, in a large population cohort study it was found that only 6% of the vaccinated population with a booster dose had a breakthrough infection approximately 6 months after vaccination [22]. However, in contrast to ours, many cases were probably missed in the above study as they did not confirm or assess the BIs based on antibody measurements [22].

BI may happen either due to the immune escape and high transmissibility of the Omicron variant, to the waning of antibodies after vaccination, or both. However, in this study, the level of antibodies after the third vaccine dose was independent from the possibility of a BI.

Several studies have assessed the contribution of different demographic and epidemiological parameters in the immune response after vaccination. The decline in IgG and NAbs up to 6 months after the second vaccine dose was not associated with age or sex in previous studies [23]. A recent Italian study showed that sex was associated with IgG levels, which were higher in females [24]. In our study, age had a significant association with TAbs-WT and NAbs-WT levels up to 8 months after the second dose and one month after the third dose. Furthermore, a sex association was found in NAbs-WT one month after the third dose, with males having higher levels.

Age has also been confirmed by many studies to affect the antibody levels [7,16]. Pozo-Balado et al. compared the immune response after the second and third doses of the vaccine at the same time points as those used in the present study between individuals >65 years old and <65 years old. In both groups the IgG antibody levels decreased over time before the booster dose, especially in the elderly, while after the booster dose a rapid increase was detected in both groups [25]. The former results are in accordance with our results, as a negative association was also found between antibody response and age. However, in our study, one month after the third dose this difference remained for TAbs-WT. In contrast, a study in India that measured the antibody response three weeks after the booster dose in more than 1000 HCWs did not find any association between immune response and age [26]. It is possible that meta-analysis studies would clarify these findings regarding the effect of age, sex, and other parameters on immune response after vaccination.

A limited number of studies have investigated the role of smoking in the immune response after vaccination. A negative association between them was found after the second BNT162b2 vaccine dose [7,27], which in the current study was found to remain for up to eight months. But, this association is lost after the third dose. Similar results were found by Yamamoto et al. [28]. However, smoking status has not been well studied in the literature, especially after the third dose, and requires further investigation.

Limitations of this study include the limited number of participants with a female predominance, and the investigation of only humoral and not cellular immunity. However, the strengths of this study were its prospective design for a long time period (16-month period) and the high number of HCWs who were included in the analysis.

## 5. Public Health Implications of the Study

Study data indicate that a significant rate of BI is detected eight months after the third dose of the BNT162b2 vaccine which is independent of the antibody response to the vaccine. These findings support the recent decision of the FDA and WHO to suggest the modification of COVID-19 vaccines to include the new XBB1.5 variant to increase vaccine effectiveness.

## 6. Conclusions

The kinetics of total and neutralizing SARS-CoV-2 antibodies indicate that breakthrough infections could be the result of waning immunity or immune evasion after the advent of the Omicron variant. Continued surveillance of the immune response and breakthrough infections in people who were vaccinated, as well as of present hybrid immunity, is necessary to guide public health decisions regarding future immunization.

## Figures and Tables

**Table 1:** Demographic and epidemiological data of vaccinated healthcare works (HCWs) with and without a breakthrough infection (BI) after administration of the second and third doses of the BNT162b2 COVID-19 vaccine.

Breakthrough Infection (BI)	After the Second Dose		After the Third Dose		Study Population *n* = 486
	No *n* = 463	Yes *n* = 23	No *n* = 294	Yes *n* = 169	
**Age (years)**	48.8 (39–56.3)	41 (31–49)	52 (42–58)	43 (35–53)	49 (38–56)
**BMI (kg/m^3^)**	24.4 (22–27.4)	23.1 (20.8–25.7)	24.4 (22–27.4)	24.3 (22–27.4)	24.4 (22–27.3)
**Sex *n* (%)**					
Male	105 (22.7)	4 (17.4)	73 (24.8)	33 (19.5)	109 (22.4)
Female	358 (77.3)	19 (82.6)	221 (75.2)	136 (80.5)	377 (77.6)
**Smoking *n* (%)**					
Yes	132 (28.5)	5 (21.7)	88 (29.9)	45 (26.6)	137 (28.2)
No	331 (71.5)	18 (78.3)	206 (70.1)	124 (73.4)	349 (71.8)
**Autoimmune disease *n* (%)**					
Yes	89 (19.2)	8 (34.8)	56 (19.0)	34 (20.1)	97 (20.0)
No	374 (80.8)	15 (65.2)	238 (81.0)	135 (79.9)	389 (80.0)
**Underlying disease *n* (%)**					
Yes	180 (38.8)	6 (26.1)	122 (41.5)	57 (33.7)	186 (38.3)
No	283 (61.2)	17 (73.9)	172 (58.5)	112 (66.3)	300 (61.7)

Notes: Age and body mass index (BMI) are expressed as median ± interquartile range.

**Table 2:** Immune response of TAbs-WT (U/mL) in vaccinated healthcare workers (HCWs) with or without a breakthrough infection (BI) 4 and 8 months after the second dose and 1 and 8 months after the third dose of the BNT162b2 vaccine.

Breakthrough Infection (BI)	TAbs-WT (U/mL)			
	After Second Dose		After Third Dose	
	4 Months (*n* = 443)	8 Months (*n* = 408)	1 Month (*n* = 386)	8 Months (*n* = 347)
**No**	*n* = 437	*n* = 397	*n* = 370	*n* = 216
	703.6 (716.1)	413.1 (479.6)	17,241.5 (14,479.0)	4441.5 (6,037.0)
**Yes**	*n* = 6	*n* = 11	*n* = 16	*n* = 131
	5,017.5 (11,185.4)	6,551.0 (14,909.0)	24,863.5 (8671.5)	25,000.0 (9,498.0)
***p*-value ^1^**	**0.022**	**<0.001**	**0.036**	**<0.001**
***p*-value ^2^**	**<0.001**			

Abbreviations: TAbs-WT—total antibodies against receptor-binding domain of wild-type SARS-CoV-2 spike protein. Values refer to median ± interquartile range; *p*-value ^1^ of Mann–Whitney U test or Kruskal–Wallis H test; *p*-value ^2^ of two-way analysis of variance (ANOVA). Statistically significant values are marked in bold.

**Table 3:** Immune response of TAbs-WT (U/mL), NAbs-WT (%), and NAbs-O (%) of vaccinated healthcare workers without a breakthrough infection at each time point.

	TAbs-WT (U/mL)				NAbs-WT (%)				NAbs-O (%)
	After Second Dose		After Third Dose		After Second Dose		After Third Dose		
	4 Months	8 Months	1 Month	8 Months	4 Months	8 Months	1 Month	8 Months	8 Months
**Sex**									
Male	*n* = 94	*n* = 85	*n* = 76	*n* = 45	*n* = 64	*n* = 61	*n* = 42	*n* = 25	*n* = 5
	659.5 (704.7)	363.4 (435.2)	20,629.5 (15,174.0)	6063.0 (6786.0)	85.5 (26.6)	56.3 (42.5)	96.9 (2.3)	97.5 (1.0)	39.7 (56.6)
Female	*n* = 393	*n* = 312	*n* = 294	*n* = 171	*n* = 231	*n* = 227	*n* = 184	*n* = 109	*n* = 109
	720.7 (713.0)	429.8 (494.1)	16,800.5 (14,019.0)	4371.0 (6104.0)	88.2 (21.0)	61.2 (35.8)	96.5 (1.8)	97.2 (1.4)	27.2 (49.5)
*p*-value ^1^	0.213	0.529	0.670	0.092	0.654	0.643	**0.016**	0.140	0.212
*p*-value ^2^	0.505				0.207				
**Age groups (years)**									
20–29	*n* = 35	*n* = 32	*n* = 25	*n* = 9	*n* = 24	*n* = 24	*n* = 15	*n* = 5	*n* = 5
	1094.0 (881.1)	716.5 (507.3)	15,287.0 (12,391.0)	6298.0 (8716.0)	89.9 (7.8)	66.5 (27.0)	96.7 (1.6)	97.6 (5.8)	11.9 (53.5)
30–39	*n* = 84	*n* = 73	*n* = 64	*n* = 22	*n* = 70	*n* = 66	*n* = 51	*n* = 19	*n* = 19
	1002.0 (803.9)	515.7 (447.7)	18,134.0 (14,387.5)	10,137.0 (16,353.0)	92.4 (10.9)	67.8 (23.3)	95.5 (1.8)	97.5 (1.4)	52.7 (63.6)
40–49	*n* = 105	*n* = 95	*n* = 85	*n* = 44	*n* = 74	*n* = 71	*n* = 56	*n* = 33	*n* = 33
	691.8 (669.5)	429.3 (362.8)	18,065.0 (11,172.0)	4683.0 (6817.0)	86.3 (23.1)	55.6 (37.4)	96.8 (1.8)	97.3 (0.8)	31.8 (52.5)
50–59	*n* = 136	*n* = 130	*n* = 133	*n* = 91	*n* = 85	*n* = 87	*n* = 72	*n* = 51	*n* = 51
	638.6 (685.3)	363.7 (393.9)	19,623.0 (13,393.0)	4371.0 (5316.0)	86.7 (23.2)	55.3 (39.9)	96.7 (1.8)	97.0 (1.4)	22.2 (44.1)
>60	*n* = 77	*n* = 67	*n* = 63	*n* = 50	*n* = 42	*n* = 40	*n* = 32	*n* = 26	*n* = 26
	483.1 (594.8)	269.0 (326.2)	12,797.0 (11,789.0)	3372.0 (5283.0)	76.1 (34.8)	45.2 (35.0)	96.8 (2.5)	97.3 (1.7)	27.2 (50.6)
*p*-value ^1^	**<0.001 ^a,b,c,d,e,f,g^**	**<0.001 ^a,b,c,d,e,g^**	**0.004 ^a,b,c^**	0.054	**<0.001 ^b,c,d,f^**	**<0.001 ^b,c,d,e^**	0.391	0.379	0.490
*p*-value ^2^	**<0.001**				**0.009**				
**Smoking**									
No	*n* = 315	*n* = 292	*n* = 272	*n* = 152	*n* = 221	*n* = 214	*n* = 167	*n* = 98	*n* = 98
	741.4 (782.2)	437.0 (494.6)	16,952.5 (14,029.5)	4441.5 (5995.5)	88.0 (20.5)	62.3 (34.3)	96.6 (1.8)	97.4 (1.0)	32.9 (50.0)
Yes	*n* = 122	*n* = 105	*n* = 98	*n* = 64	*n* = 74	*n* = 74	*n* = 59	*n* = 36	*n* = 36
	623.3 (674.7)	327.3 (420.7)	18,003.5 (14,715.0)	4375.0 (6028.5)	85.2 (23.3)	50.8 (35.4)	96.8 (1.7)	97.0 (1.7)	17.6 (50.0)
*p*-value ^1^	**0.012**	**0.009**	0.919	0.813	**0.049**	**0.003**	0.477	0.620	0.180
*p*-value ^2^	0.999				0.073				
**Autoimmune diseases**									
No	*n* = 353	*n* = 318	*n* = 296	*n* = 174	*n* = 242	*n* = 236	*n* = 181	*n* = 108	*n* = 108
	716.2 (690.8)	419.1 (457.9)	17,396.5 (14,534.0)	4550.5 (7490.0)	87.8 (20.9)	61.3 (36.2)	96.7 (1.8)	97.4 (1.1)	34.9 (52.9)
Yes	*n* = 84	*n* = 79	*n* = 74	*n* = 42	*n* = 53	*n* = 52	*n* = 45	*n* = 26	*n* = 26
	643.3 (811.0)	385.3 (547.9)	16,692.0 (13,465.0)	3518.0 (4475.0)	87.0 (30.6)	58.2 (36.3)	96.5 (1.9)	97.1 (2.2)	15.9 (31.8)
*p*-value ^1^	0.130	0.272	0.937	**0.043**	0.289	0.264	0.932	0.232	**0.049**
*p*-value ^2^	**0.029**				0.154				
**Underlying diseases**									
No	*n* = 268	*n* = 244	*n* = 219	*n* = 117	*n* = 178	*n* = 173	*n* = 122	*n* = 73	*n* = 73
	747.4 (767.5)	438.5 (497.8)	16,469.0 (14,666.0)	4448.0 (8411.0)	87.9 (18.4)	62.2 (30.0)	96.5 (1.9)	97.2 (1.4)	24.7 (52.4)
Yes	*n* = 169	*n* = 153	*n* = 151	*n* = 99	*n* = 117	*n* = 115	*n* = 104	*n* = 61	*n* = 61
	679.5 (689.6)	398.6 (464.0)	18,065.0 (13,643.0)	4435.0 (5608.0)	86.7 (25.9)	55.3 (39.9)	96.7 (1.8)	97.4(1.2)	32.1(45.5)
*p*-value ^1^	0.283	0.071	0.307	0.755	0.125	0.054	0.090	0.488	0.610
*p*-value ^2^	0.151				0.145				

Abbreviations: TAbs-WT—total antibodies against receptor-binding domain of wild-type SARS-CoV-2 spike protein; NAbs-WT—neutralizing antibodies against receptor-binding domain of wild-type SARS-CoV-2 spike protein (%); NAbs-O—neutralizing antibodies against receptor-binding domain of Omicron SARS-CoV-2 spike protein (%). Values refer to median ± interquartile range; *p*-value ^1^ of Mann–Whitney U test or Kruskal–Wallis H test; *p*-value ^2^ of two-way analysis of variance (ANOVA). Statistically significant values are marked in bold. Differences using post hoc Tukey b test were found between ^a^ 60+ and 40–49 years, ^b^ 60+ and 30–39 years, ^c^ 60+ and 20–29 years, ^d^ 50–59 and 30–39 years, ^e^ 50–59 and 20–29 years, ^f^ 40–49 and 30–39 years, ^g^ 40–49 and 20–29 years.

## Data Availability

All relevant data are published within the paper.

## Supplementary Materials

The following supporting information can be downloaded at: https://www.mdpi.com/article/10.3390/microorganisms11082010/s1, Figure S1: CONSORT flow diagram of the study; Table S1: STROBE checklist of this prospective cohort study; Table S2: Adverse events after the third dose of BNT162b2 COVID-19 vaccine in total study population of healthcare workers (HCWs) and in each category depending on breakthrough infection (BI); Table S3: Median values of TAbs-WT (U/mL) SARS-CoV-2 spike antibodies after 4 and 8 months after the second dose and 1 and 8 months after the third dose of BNT162b2 vaccine in HCWs with a breakthrough infection.
