# Supplementary material for: Kinetics of SARS-CoV-2 Spike Antibodies after the Second and Third Dose of the BNT162b2 COVID-19 Vaccine and Association with Epidemiological Characteristics and Breakthrough Infection in a Cohort Study of Healthcare Workers
Source: Microorganisms. 2023 Aug 4;11(8):2010. doi: 10.3390/microorganisms11082010 (PMC10458561; doi:10.3390/microorganisms11082010)
Supplement: Supplementary file 1 [file microorganisms-11-02010-s001.zip › microorganisms-2521344-supplementary.pdf]

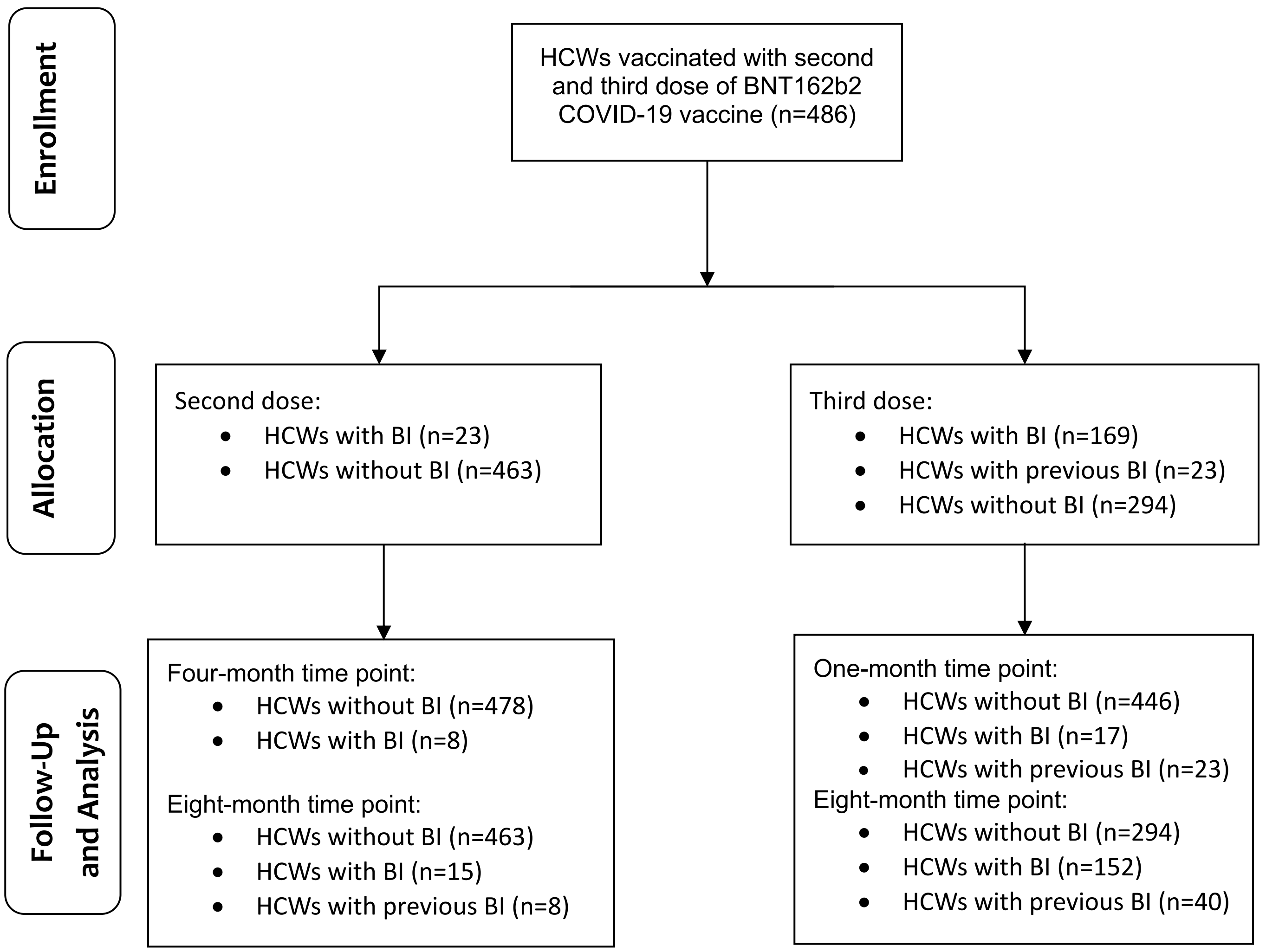

Figure S1. CONSORT flow diagram of the study.

Table S1. STROBE checklist of this prospective cohort study.

	Item No.	Recommendation	Page No.	Relevant lines
Title and abstract	1	(a) Indicate the study's design with a commonly used term in the title or the abstract	1	5 and 18
		(b) Provide in the abstract an informative and balanced summary of what was done and what was found	1	13-27
Introduction				
Background/rationale	2	Explain the scientific background and rationale for the investigation being reported	1-2	31-58
Objectives	3	State specific objectives, including any prespecified hypotheses	2	59-63
Methods				
Study design	4	Present key elements of study design early in the paper	2	66-88
Setting	5	Describe the setting, locations, and relevant dates, including periods of recruitment, exposure, follow-up, and data collection	2	66-81
Participants	6	Cohort study—Give the eligibility criteria, and the sources and methods of selection of participants. Describe methods of follow-up	2	66-81
Variables	7	Clearly define all outcomes, exposures, predictors, potential confounders, and effect modifiers. Give diagnostic criteria, if applicable	2	82-88
Data sources/ measurement	8	For each variable of interest, give sources of data and details of methods of assessment (measurement). Describe comparability of assessment methods if there is more than one group	2-3	90-109
Quantitative variables	11	Explain how quantitative variables were handled in the analyses. If applicable, describe which groupings were chosen and why	3	111-120
Statistical methods	12	(a) Describe all statistical methods, including those used to control for confounding	3	111-120
		(b) Describe any methods used to examine subgroups and interactions	3	111-120

Continued on next page

Participants	13	(a) Report numbers of individuals at each stage of study—eg numbers potentially eligible, examined for eligibility, confirmed eligible, included in the study, completing follow-up, and analysed	3-4	123-156
		(b) Consider use of a flow diagram	2	75
Descriptive data	14	(a) Give characteristics of study participants (eg demographic, clinical, social) and information on exposures and potential confounders	3-4	123-156
Outcome data	15	Cohort study—Report numbers of outcome events or summary measures over time	4-6	158-199
Other analyses	17	Report other analyses done—eg analyses of subgroups and interactions, and sensitivity analyses	6-8	222-239
Key results	18	Summarise key results with reference to study objectives	8-9	242-307
Limitations	19	Discuss limitations of the study, taking into account sources of potential bias or imprecision. Discuss both direction and magnitude of any potential bias	9	308-309
Interpretation	20	Give a cautious overall interpretation of results considering objectives, limitations, multiplicity of analyses, results from similar studies, and other relevant evidence	8-9	242-312
Generalisability	21	Discuss the generalisability (external validity) of the study results	9	315-318

Table S2. Adverse events (AEs) after the third dose of BNT162b2 COVID-19 vaccine in the total study population of healthcare workers (HCWs) and in each group according to whether they had breakthrough infection (BI).

Adverse events	Total n=462	No BI vaccinated HCWs n=279	HCWs with BI after the 3rd dose n=165	HCWs with BI before the 3rd dose n=18
**Local n (%)**				
Pain	294 (63.6)	177 (63.4)	105 (63.6)	13 (72.2)
Edema	28 (6.1)	17 (6.1)	10 (6.1)	1 (5.6)
Erythema	15 (3.2)	9 (3.2)	6 (3.6)	0 (0.0)
Pruritus	3 (0.7)	2 (0.7)	1 (0.6)	0 (0.0)
Number of Local AEs	4	4	4	2
**Systemic n (%)**				
Fatigue	135 (29.2)	86 (30.8)	47 (28.5)	3 (16.7)
Lymphadenopathy	85 (18.4)	52 (18.6)	31 (18.8)	2 (11.1)
Myalgias/Arthralgias	72 (15.6)	47 (16.8)	23 (13.9)	1 (5.6)
Headache	51 (11.0)	31 (11.1)	19 (11.5)	3 (16.7)
Fever >38.5oC	36 (7.8)	23 (8.2)	15 (9.1)	0 (0.0)
Chills	36 (7.8)	18 (6.5)	18 (10.9)	1 (5.6)
Drowsiness	17 (3.7)	9 (3.2)	7 (4.2)	1 (5.6)
Dizziness	11 (2.4)	6 (2.2)	5 (3.0)	1 (5.6)
Chest pain	5 (1.1)	4 (1.4)	0 (0.0)	0 (0.0)
Diarrhea	4 (0.9)	1 (0.4)	3 (1.8)	0 (0.0)
Nasal congestion	3 (0.7)	2 (0.7)	0 (0.0)	1 (5.6)
Sore throat	3 (0.7)	2 (0.7)	1 (0.6)	0 (0.0)
Neck pain	2 (0.4)	2 (0.7)	0 (0.0)	0 (0.0)
Formicary	2 (0.4)	2 (0.7)	0 (0.0)	0 (0.0)
Hypertension	2 (0.4)	2 (0.7)	0 (0.0)	0 (0.0)
Dyspnea	2 (0.4)	0 (0.0)	2 (1.2)	0 (0.0)
Vision disorders	2 (0.4)	2 (0.7)	0 (0.0)	0 (0.0)
HSV resurgence	2 (0.4)	1 (0.4)	1 (0.6)	0 (0.0)
Cough	1 (0.2)	1 (0.4)	0 (0.0)	0 (0.0)
Tachycardia	1 (0.2)	1 (0.4)	0 (0.0)	0 (0.0)
Hypotension	1 (0.2)	1 (0.4)	0 (0.0)	0 (0.0)
Metallic taste	1 (0.2)	0 (0.0)	1 (0.6)	0 (0.0)
Tinnitus with temporary hearing loss	1 (0.2)	1 (0.4)	0 (0.0)	0 (0.0)
Number of Systemic AEs	23	21	13	8

Table S3. TAbs-WT (U/ml) SARS-CoV-2 spike antibodies in 4 and 8 months after the second dose and 1 and 8 months after the third dose of BNT162b2 vaccine in HCWs with a breakthrough infection.

	TAbs-WT (U/ml)			
	After 2nd dose		After 3rd dose	
	4 months	8 months	1 month	8 months
**Sex**				
Male			n=3	n=23
			10,377.0 (19,172.0)	25,000.0 (6,320.0)
Female	n=6	n=11	n=13	n=108
	5,017.5 (11,185.4)	6,551.0 (14,909.0)	25,000.0 (5,643.0)	24,724.0 (10,293.5)
p-value¹			0.151	0.283
p-value²	**0.027**			
**Age groups (years)**				
20-29		n=1	n=1	n=14
		17,473.0 (0.0)	25,000.0 (0.0)	25,000.0 (8,271.0)
30-39	n=3	n=4	n=2	n=29
	8,392.0 (11,510.4)	4,981.0 (2,801.5)	15,125.5 (8,463.0)	24,191.0 (11,495.0)
40-49	n=2	n=3	n=6	n=37
	6,907.5 (10,529.0)	6,551.0 (8,324.7)	25,000.0 (273.0)	24,149.0 (7,913.0)
50-59	n=1	n=3	n=5	n=41
	202.7 (0.0)	24,563.0 (22,436.0)	24,008.0 (8,567.0)	25,000.0 (9,498.0)
>60			n=2	n=10
			15,414.0 (19,172.0)	24,012.0 (5,535.0)
p-value¹	0.343	0.392	0.425	0.828
p-value²	0.258			
**Smoking**				
No	n=5	n=6	n=11	n=96
	1,643.0 (7,405.4)	7,211.0 (12,363.0)	24,727.0 (14,106.0)	25,000.0 (8,961.0)
Yes	n=1	n=5	n=5	n=35
	12,497.0 (0.0)	4,852.0 (6,723.0)	25,000.0 (5,643.0)	24,448.0 (13,367.0)
p-value¹	0.143	0.715	0.468	0.399
p-value²	0.490			
**Autoimmune diseases**				
No	n=4	n=6	n=13	n=104
	6,907.5 (11,019.7)	5,701.5 (5,345.0)	25,000.0 (5,643.0)	25,000.0 (9,318.5)
Yes	n=2	n=5	n=3	n=27
	4,297.4 (8,189.3)	9,287.0 (19,453.0)	16,433.0 (14,106.0)	23,827.0 (9,510.0)
p-value¹	0.355	0.201	0.472	0.553
p-value²	0.326			
**Underlying diseases**				
No	n=4	n=6	n=12	n=90
	10,282.0 (7,645.2)	5,830.5 (3,019.0)	25,000.0 (7,209.5)	25,000.0 (11,973.0)
Yes	n=2	n=5	n=4	n=41
	922.9 (1,440.3)	9,287.0 (21,999.0)	20,220.5 (1,1099.0)	25,000.0 (7,913.0)
p-value¹	0.165	0.584	0.331	0.424
p-value²	0.280			

Abbreviations: TAbs-WT; Total antibodies to Receptor Binding Domain of SARS-CoV-2 spike protein. Values refer to median ± interquartile range, *p*-value¹ of Mann-Whitney U test or Kruskall-Wallis H test, *p*-value² of two-way analysis of variance (ANOVA). Statistically significant values are marked in bold.
